# Genome-Wide Screening and Functional Analysis Reveal That the Specific microRNA nlu-miR-173 Regulates Molting by Targeting Ftz-F1 in *Nilaparvata lugens*

**DOI:** 10.3389/fphys.2018.01854

**Published:** 2018-12-20

**Authors:** Jie Chen, Teng Chao Li, Rui Pang, Xiang Zhao Yue, Jian Hu, Wen Qing Zhang

**Affiliations:** State Key Laboratory of Biocontrol, School of Life Sciences, Sun Yat-sen University, Guangzhou, China

**Keywords:** nlu-miR-173, *NlFtz-F1*, molting, *Nilaparvata lugens*, pest control

## Abstract

**Background:** Molting is a crucial physiological behavior during arthropod growth. In the past few years, molting as well as chitin biosynthesis triggered by molting, is subject to regulation by miRNAs. However, how many miRNAs are involved in insect molting at the genome-wide level remains unknown.

**Results:** We deeply sequenced four samples obtained from nymphs at the 2nd−3rd and 4th−5th instars, and then identified 61 miRNAs conserved in the Arthropoda and 326 putative novel miRNAs in the brown planthopper *Nilaparvata lugens*, a fearful pest of rice. A total of 36 mature miRNAs with significant different expression levels at the genome scale during molting, including 19 conserved and 17 putative novel miRNAs were identified. After comparing the expression profiles, we found that most of the targets of 36 miRNAs showing significantly differential expression were involved in energy and hormone pathways. One of the 17 putative novel miRNAs, nlu-miR-173 was chosen for functional study. nlu-miR-173 acts in 20-hydroxyecdysone signaling through its direct target, *N. lugens Ftz-F1*(*NlFtz-F1*), a transcription factor. Furthermore, we found that the transcription of nlu-miR-173 was promoted by Broad-Complex (*BR-C*), suggesting that its involvement in the 20-hydroxyecdysone pathway contributes to proper molting function.

**Conclusion:** We provided a comprehensive resource of miRNAs associated with insect molting and identified a novel miRNA as a potential target for pest control.

## Introduction

Insects which are the hugest group of animals sometimes are human disease vectors and even more agricultural pests in the nature (Zhang et al., 2009; Liu et al., 2018). Over thousands of years, humans have developed necessary measures to prevent disease and pests from ourselves and plants. Although pesticides have played a pivotal role in both agriculture and public health, their widespread use has also been linked to the development of insecticide resistance and environmental issues (Soko et al., 2015). Thus, pest control urgently requires seeking novel types of pesticides such as insect growth regulators or novel targets in insects for pesticide development. Molting is an important developmental behavior during arthropod growth, and the genes involved in insect molting are usually used as effective targets for pest control (Retnakaran et al., 2003; Guerrero and Rosell, 2005; Soko et al., 2015; Niwa and Niwa, 2016). Chitin, the second most rich polysaccharide in biomass after cellulose (Tharanathan and Kittur, 2003; Kurita, 2006; Zakrzewski et al., 2014), is one of the most important substances during molting (Zhu et al., 2016). In recent years, microRNAs have been discovered to be involved in various developmental processes of animals. However, little miRNAs have been reported to act in insect molting or chitin biosynthesis (Belles, 2017). In *Drosophila melanogaster*, two miRNA family, Let-7-Complex and miR-34 function as a key factor to control insect metamorphosis (Sempere et al., 2003). In *Bombyx mori*, miRNAs with intact expression profiles during the whole life cycle have been established (Liu et al., 2009; Pan et al., 2017). In *Helicoverpa armigera*, miR-24 shows a negative correlation with chitinase transcript expression lavel in fifth-instar larvae (Agrawal et al., 2013). In *Nilaparvata lugens*, The conserved miRNAs miR-8-5p and miR-2a-3p targets two genes in the chitin biosynthesis pathway responsed by 20-hydroxyecdysone signaling (Chen et al., 2013). Chitin synthase A repressed by microRNA and dsRNA injection *in vivo* shows to be good for pest control (Li et al., 2017). These results indicate that the miRNAs involved in insect molting are largely unknown.

The brown planthopper *N. lugens* is one fearful pest of rice. Since its genome was published in 2014 (Xue et al., 2014), *N. lugens* has become a model hemipteran insect for scientific study such as migration, genetics, insecticide resistance, and adaptive evolution in insects. In this study, we provide the first resource for the dynamic profiles of miRNAs during insect molting, and we identify a novel miRNA, nlu-miR-173, that regulates insect molting by targeting the transcription factor *NlFtz-F1*.

## Materials and Methods

### Insects and Cells

The brown planthopper *N. lugens* used in our work has been reared steadily on susceptible rice varieties in our laboratory (Chen et al., 2013).

*Drosophila* S2 cells were cultured in Schneider's medium (Gibco) supplemented with 10% fetal bovine serum at 27 °C.

### miRNA Sequencing and Prediction

Solexa sequencing was performed as described previously by BGI (Shenzhen, China) (Chen et al., 2013). Eighteen or more nucleotides sequences were blasted into the genome of *N. lugens* (version 6.1) with their entire length by SOAP (Li et al., 2008). First, miRBase miRNA Database (v20.0) was used for known miRNAs identification by BLAST search. Then, other sequences which have hairpin structures can be used to predict novel miRNAs using MIREAP by genome mapping.

### Target Genes Prediction

We predicted targets for 36 miRNAs using both miRanda and RNAhybrid programs by a same criterion that six or more bases perfectly matched in the seed region (2–7 nt). In addition, higher filters were required by RNAhybrid, for example, free energy should be < -25 and the 3′ ends of sequences should have complementary bases.

### Semi-quantitative and Quantitative PCR of miRNAs

The expression levels of 40 predicted miRNAs during the molting of 2nd−3rd and 4th−5th instars were detected by semi-quantitative PCR. The forward primers was miRNAs themselves and the reverse primer was a linker primer according to the specification of mir-X™ miRNA first-strand synthesis kit (TaKaRa, Japan).

The expression levels of *Ftz-F1*, nlu-mir-173, *BR-C*, and *E74* (Ecdysone-induced protein 74EF). **Figures 4**, **7** were detected by qPCR using LightCycler 480 system. All the primers details were shown in Table S5. *U6* was used as an internal control for miRNA assay and *Actin* was for gene assay. SYBR Premix Ex Taq was used (TaKaRa, Japan).

### Co-expression of miRNAs and Their Targets in S2 Cells

We use pGL3-Basic and pRL-null vectors modified by the *Pac* promoter to detect luciferase signals in S2 cells. Details were related similarly to the previous work (Chen et al., 2013).

### Promoter Assay

The promoter assay was related similarly to the previous work (Chen et al., 2013).

### 20E Assay

An Ecdysone ELISA Kit (Biosense, China) was used to quantify the content of ecdysteroids. Ten individuals per sample were selected and considered one set for the ecdysteroid detection. Insects were cleaned, weighed and homogenized in 70% methanol. Supernatants were collected by 12,000 g for 10 min at 4°C, and the pellets were repeated twice aboved. Supernatants overall were dried and resoluted between 70% methanol and hexane to remove non-polar lipids. The hexane phase was throwed and the lower methanolic phase was dried, then re-dissolved in 100 μl methanol.

### Feeding and Injection on Nymphs

The feeding and injection experiments were improved by our laboratory. The suitable glass capillary (1B100F-4, WPI, USA) was used to injected into the abdomen of early fifth-instar adults which was on narcosis by CO_2_. Fifty nanoliters of liquid was injected in our experiments. In the feeding experiment, we placed the same age of third-instar individuals into a chamber for miRNA mimics or inhibitor feeding. After 7 days with continuous feeding, the survival rates were recorded. Other details were related similarly to the previous work (Chen et al., 2013).

### Chitin Content Analysis

The method of assay was amended slightly on the basis of that mentioned by Arakane et al. (2004).

### Statistical Analysis

The asterisk indicated the significant differences between control and treatment groups by *t*-test. Significant differences were marked by letters from different groups of survival rates (*p* < 0.05, LSR, SPSS). In qPCR assay, each bar indicates the mean ± SEM with three independent experiments, and asterisks indicate significant differences at the *p* < 0.05 level.

## Results

### Deep-sequencing of microRNAs in *N. lugens*

Instar transition is the best period to detect the dynamic changes in the miRNA levels during molting. Four time periods, the end of the 2nd instar (E2nd), the beginning of the 3rd instar (B3rd), the end of the 4th instar (E4th) and the beginning of the 5th instar (B5th), were used to represent two periods of molting, namely, the 2nd−3rd and 4th−5th molting periods. *N. lugens* individuals at the above four periods were chosen and sequenced for their small RNAs using Solexa high-throughput. After removing unrelated data, we obtained 11,869,784, 11,883,255, 11,891,547, and 11,873,764 clean reads from the E2nd, B3rd, E4th, and B5th larvae, respectively (Table 1). Using a filtration series, we finally obtained 541,980, 399,807, 469,416, and 511,065 small RNA sequences, respectively. These were used as candidates for identifying known miRNAs by mapping with miRNA database in miRBase (Table 1). At last, we identified 619, 581, 616, and 557 conserved miRNA sequences in the four samples, respectively. The others were further mapped to the *N. lugens* genome to predict novel miRNAs. We obtained 183, 158, 151, and 168 putative novel miRNAs (Table S1). In total, we found 61 miRNA families conserved in the Arthropoda (Table S1), and 326 putative novel miRNAs at the genome-wide scale in *N. lugens*.

**Table 1:** Statistics of small RNA sequences from the individual libraries.

	**E2nd**	**B3rd**	**E4th**	**B5th**
Total reads	11,942,246 (100%)	11,945,775 (100%)	11,946,938 (100%)	11,939,071 (100%)
Clean reads^a^	11,869,784 (99.39%)	11,883,255 (99.48%)	11,891,547 (99.54%)	11,873,764 (99.45%)
Sequences of 18–30 nt^a^	11,832,987 (99.08%)	11,861,865 (99.30%)	11,823,765 (98.97%)	11,867,827 (99.40%)
matched to the genome^b^	9,068,122 (76.40%)	9,035,503 (76.04%)	8,911,769 (74.94%)	9,215,165 (77.61%)
rRNA^b^	94,496 (0.80%)	85,456 (0.72%)	382,460 (3.22%)	104,619 (0.88%)
tRNA^b^	222,631 (1.88%)	210,797 (1.77%)	413,570 (3.48%)	74,917 (0.63%)
snRNA^b^	48,686 (0.41%)	48,686 (0.41%)	48,686 (0.41%)	48,686 (0.41%)
snoRNA^b^	91 (0.00%)	117 (0.00%)	222 (0.00%)	102 (0.00%)
Exon_antisense^b^	2,226,765 (18.76%)	2,184,584 (18.38%)	1,957,306 (16.46%)	2,345,765 (19.76%)
Exon_sense^b^	670,758 (5.65%)	619,568 (5.21%)	597,734 (5.03%)	697,369 (5.87%)
Intron_antisense^b^	1,493,539 (12.58%)	1,563,384 (13.16%)	1,521,521 (12.79%)	1,493,136 (12.58%)
Intron_sense^b^	1,365,686 (11.51%)	1,411,455 (11.88%)	1,358,092 (11.42%)	1,341,692 (11.30%)
Repeat small RNAs^b^	698,767 (5.89%)	730,695 (6.15%)	707,346 (5.95%)	716,879 (6.04%)
Known miRNAs^b^	541,980 (4.57%)	399,807 (3.36%)	469,416 (3.95%)	511,065 (4.30%)
Unannotated small RNAs^b^	4,506,385 (37.97%)	4,627,928 (38.94%)	4,424,972 (37.21%)	4,539,636 (38.23%)

a *The percentage of the total reads*.

b *The percentage of the clean reads*.

### Differentially Expressed miRNAs During Molting

**Figure 1:**
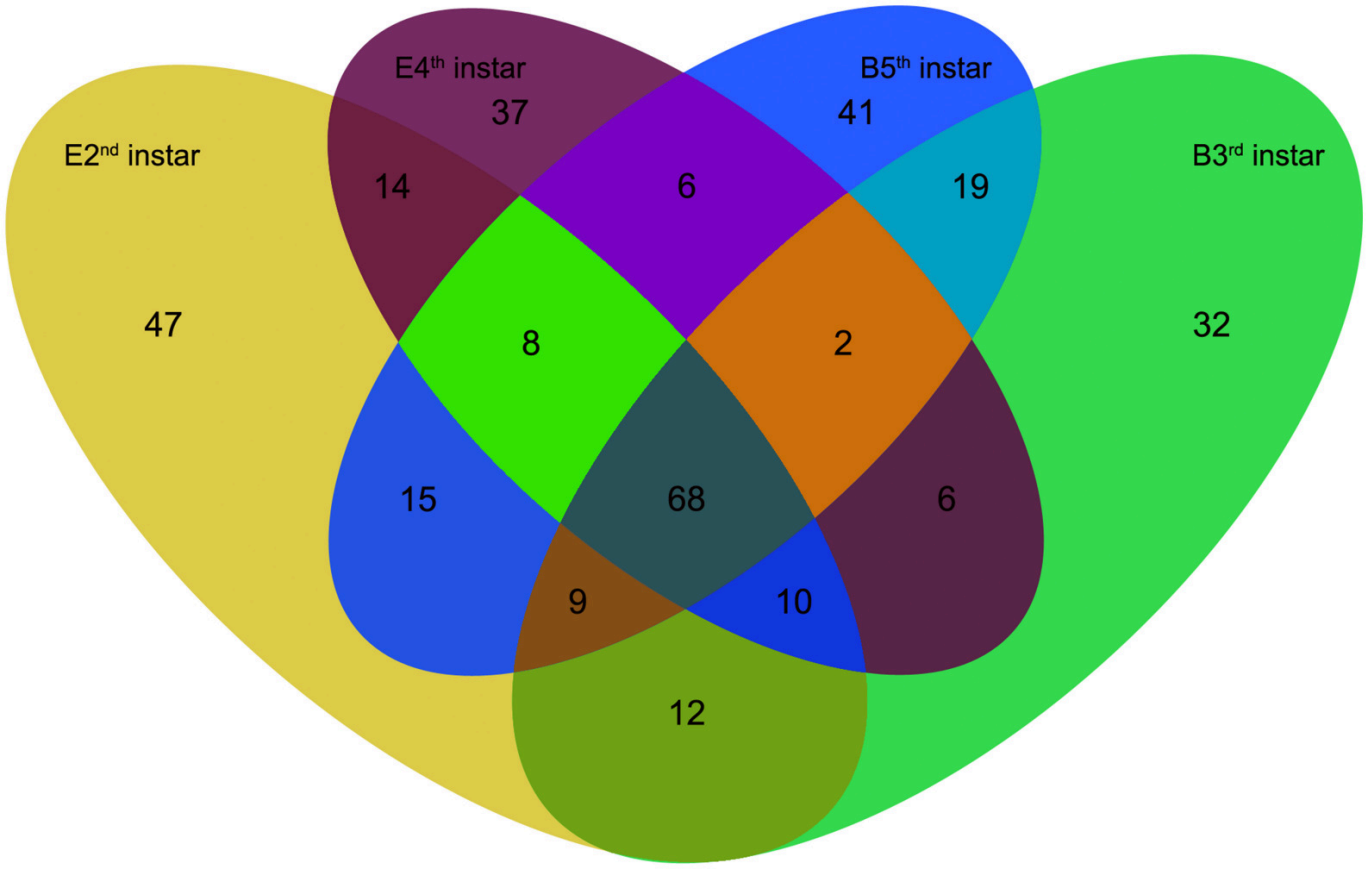
The numbers of putative novel miRNAs in four samples of *N. lugens*. E2nd instar, the miRNA numbers acquired from the sample at the end of the 2nd instar; B3rd instar, the miRNA numbers acquired from the sample at the beginning of the 3rd instar; E4th instar, the miRNA numbers acquired from the sample at the end of the 4th instar; B5th instar, the miRNA numbers acquired from the sample at the beginning of the 5th instar. The overlapping numbers indicate the common miRNAs in different samples.

**Figure 2:**
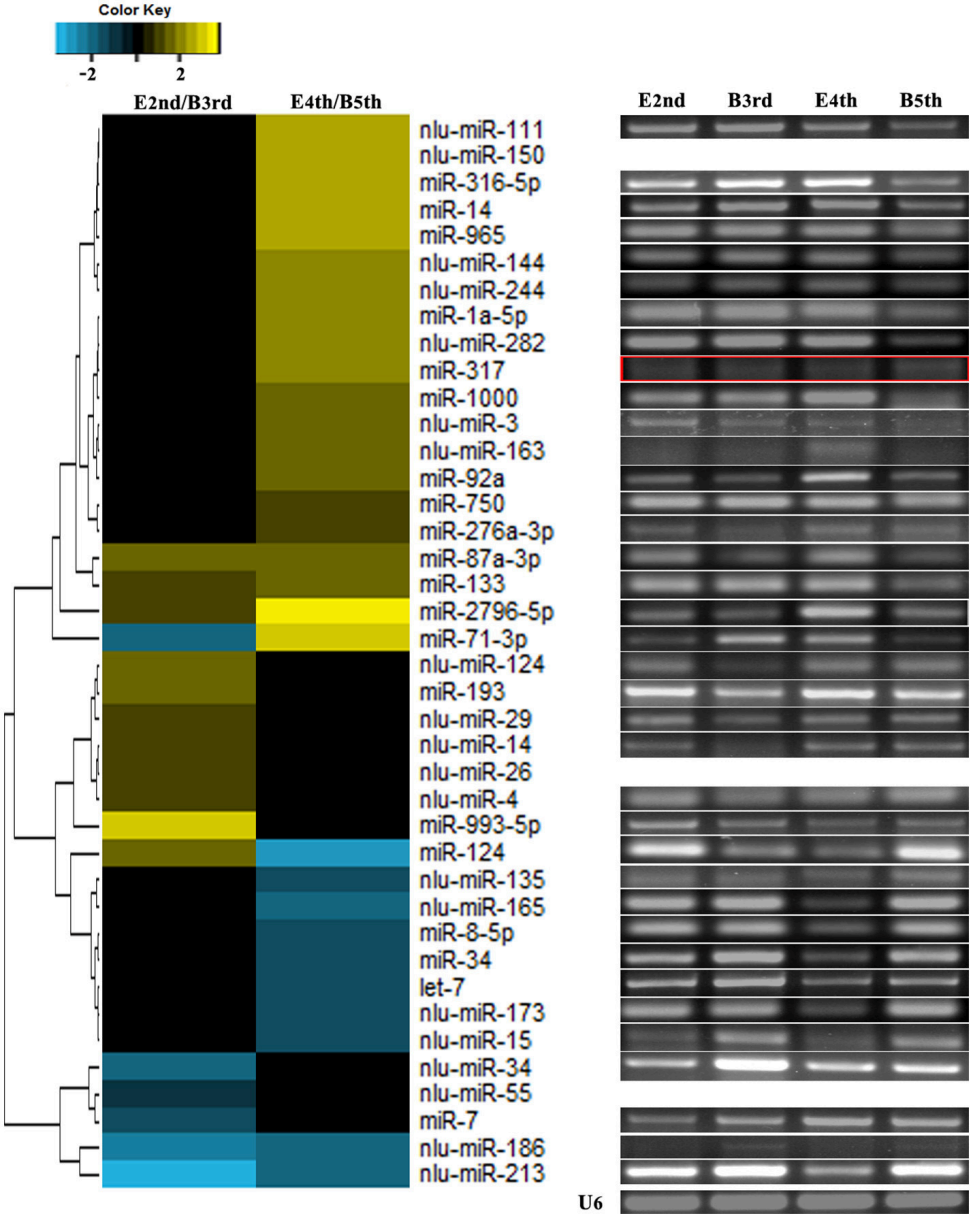
Validation of expression changes of miRNAs during molting. The left heatmap indicates the sequencing data of miRNA expression ratios. The bar represents the scale of the ratios of expression levels (log 2). The right electrophoretograms indicate the semi-quantitative PCR of miRNAs, using U6 as an internal control. E2nd/B3rd, the expression level in the sample at the end of the 2nd instar/the expression level in the sample at the beginning of the 3rd instar; E4th/B5th, the expression level in the sample at the end of the 4th instar/the expression level in the sample at the beginning of the 5th instar. The empty and red boxes indicate false positive results.

We compared the expression levels of putative novel miRNA profiles during the 2nd−3rd and 4th−5th instar molting periods (Figure 1) and found 20 putative novel miRNAs with significantly different expression in larval molting of *N. lugens* by the criterion of log2 defined as having a ratio of absolute values ≥1.0 (Figure 2). In addition, 20 miRNAs that are conserved in arthropods were also identified (Figure 2). As shown in the profiles, only 7 miRNAs (miR-87a-3p, miR-133, miR-2796-5p, miR-71-3p, miR-124, nlu-miR-186, and nlu-miR-213) had different expression levels during both molting periods. Furthermore, more miRNAs showed differential expression during the 4th−5th molting period, which may be due to increased complexity of the physiological activities during this period. To confirm the expression changes of 40 miRNAs determined using Solexa sequencing, an independent experiment using semi-quantitative PCR was performed. The results showed that only four miRNAs were unsuccessfully cloned (miR-317, nlu-miR-150, nlu-miR-26, and nlu-miR-55) (Figure 2), indicating a strong consistency between the fold-change values obtained via sequencing and the semi-quantitative PCR.

### Potential Targets of miRNAs Involved in Molting

We predicted target genes for 36 miRNAs using both miRanda and RNAhybrid except for four false positive miRNAs, and 173 targets for 19 conserved miRNAs and 108 targets for 17 putative novel miRNAs with high filters both in two programs were found (Table S2). In total, 122 genes possibly targeted by the identified 36 miRNAs were annotated in help of the genome, with an average of 3.4 targets per miRNA. A high amount of the potential targets were involved in signal transduction, cellular structure, transcription, and translation. Moreover, targets involved in cuticular and hormone regulation, energy and other metabolic pathways were overrepresented (Table S3).

Because target prediction can produce false positives, RNA sequencing was also used to determine the gene expression patterns at the four time periods (E2nd, B3rd, E4th, and B5th). Using the transcriptome data of *N. lugens* (data not shown), 539 unigenes demonstrated lower expression levels at the beginning of the 3rd instar (B3rd) than at the end of the 2nd instar (E2nd), whereas 211 unigenes were up-regulated at B3rd compared to B2nd. Similarly, 841 unigenes demonstrated lower expression levels at the beginning of the 5th instar (B5th) than at the end of the 4th instar (E4th), whereas 830 unigenes were up-regulated at B5th compared to E4th. To exclude the possibility that different genes participate in molting at different instars, we chose genes that were differentially expressed during both time periods. A total of 82 unigenes up-regulated and 414 unigenes down-regulated during molting were identified at the whole-genome level. Furthermore, Gene Ontology (GO) abundance analysis of these genes revealed terms related to catalytic activity (Figure S1A), oxidation-reduction processes (Figure S1B), and structural molecule activity, including the structural constituents of the chitin-based cuticle, that were all statistically significant (Figure S1C).

**Figure 3:**
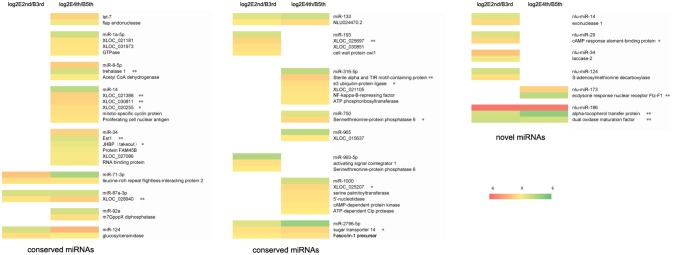
Expression profiles of miRNAs and their targets during molting. The bar represents the scale of ratios of expression levels (log 2). E2nd/B3rd, the expression level in the sample at the end of the 2nd instar/the expression level in the sample at the beginning of the 3rd instar; E4th/B5th, the expression level in the sample at the end of the 4th instar/the expression level in the sample at the beginning of the 5th instar. The genes behind the miRNA indicate that these are the potential targets of this miRNA. A single asterisk indicates a significant difference at the *p* < 0.05 level. A double asterisk indicates a significant difference at the *p* < 0.01 level.

We chose negative correlations between miRNAs and targets for further study through small RNA sequencing and RNA sequencing (Figure 3, Table S4). Most of the targets showing significant differential expression (defined as having a ratio of absolute values ≥1.5) were involved in energy and hormone functions. For example, trehalase was a target of miR-8-5p and is a link between the hormone and chitin biosynthesis pathways according to our previous work (Chen et al., 2013). In total, we identified a total of 12 miRNAs (let-7, miR-34, miR-1000, miR-133, miR-14, miR-71-3p, miR-965, miR-87, miR-8, nlu-miR-213, nlu-miR-186, and nlu-miR-173) and target molecules showing significantly different expression levels as potential factors during molting. Among the above potential miRNAs involved in insect molting, we chose nlu-miR-173 for functional study because of its target (*NlFtz-F1*) and species specificity.

### *NlFtz-F1* Is a Target of nlu-miR-173 in Response to 20-hydroxyecdysone Signaling

**Figure 4:**
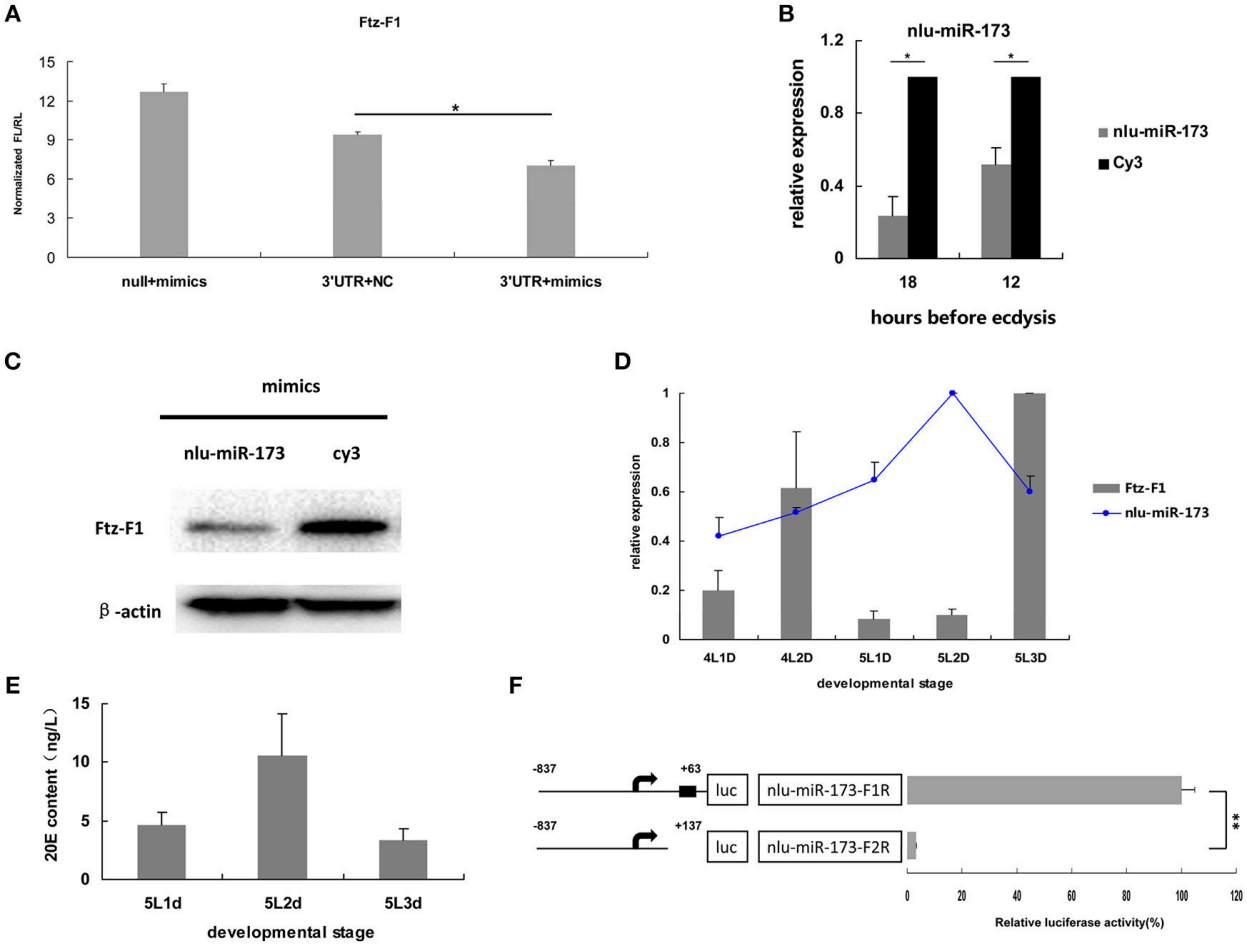
*NlFtz-F1* is a target of nlu-miR-173 in response to 20-hydroxyecdysone signaling. **(A)** Co-transfection of nlu-miR-173 and its target *NlFtz-F1*. NC represents an unrelated small RNA sequence, and null represents a reporter without the 3′ UTR sequence. A single asterisk indicates a significant differences between the control group and the treated group at the *p* < 0.05 level. Each point represents the mean ± SEM from three independent experiments. **(B)** Transcription analysis. All mRNA levels are shown relative to the level of β*-actin*. A single asterisk indicates a significant difference between the two groups at the *p* < 0.05 level. Each point represents the mean ± SEM (*n* = 3). **(C)** Western blot. Protein extracts (5 μg) from the injected nymphs were loaded onto 12% SDS-PAGE gels. The gels were immunostained with anti-Ftz-F1 serum. β-Actin was used as a control. **(D)** Expression levels of nlu-miR-173 and *NlFtz-F1*. 4L1D: Nymphs on the 1st day of the 4th instar. All miRNA levels are shown relative to the *U6* level, all mRNA levels are expressed relative to the level of β-actin, and the expression level of nlu-miR-173 is normalized to 5L2D, and that of *NlFtz-F1* is normalized to 5L3D. **(E)** 20E content. **(F)** Luciferase assays used to test the *BR-C* binding sites in the promoters of nlu-miR-173. Black boxes means binding sites for *BR-C*. Each point represents the mean ± SEM from three independent experiments. A double asterisk indicates a significant difference between the 2 days at the *p* < 0.01 level. The highest luciferase level of a segment is designated as 100%.

To validate *NlFtz-F1* as a target of nlu-miR-173, the expression of luciferase from the constructs bearing the 3′ UTR sequences of *NlFtz-F1* was detected after co-transfection of nlu-miR-173 and *NlFtz-F1* in cells. Because of unavailable cell lines in *N. lugens*, we use S2 cells for identification between miRNA and its target referring to the previous work in Chen et al. (2013). The construct with co-transfection of nlu-miR-173 decreased by 44.60%, while the construct without co-transfection of nlu-miR-173 also showed a little reduced expression of luciferase relative to the construct without the 3′ UTR sequences co-expressed with nlu-miR-173(Figure 4A). This indicated that the 3′ UTR sequences of *NlFtz-F1* may include other seed regions for other miRNAs. *In vivo*, the transcription level (Figure 4B) and protein level (Figure 4C) of *NlFtz-F1* were decreased after mimics of nlu-miR-173 injection, confirmed that *NlFtz-F1* was the target of nlu-miR-173.

Besides, the expression levels of nlu-miR-173 and *NlFtz-F1* were detected *in vivo* using qPCR. The results showed that the expression level of nlu-miR-173 decreased largely on the last day and increased on the previous day during the 5th instar, whereas the target gene *NlFtz-F1* showed the opposite pattern (Figure 4D). Combined with the similar expression pattern of 20E (Figure 4E), these findings suggested that *NlFtz-F1* and nlu-miR-173 may be in response to 20-hydroxyecdysone signaling. The transcription factor *BR-C* is a key gene in 20-hydroxyecdysone signaling. We use several programs to analyze whether the promoter of nlu-miR-173 was regulated by *BR-C*. The luciferase activity assay showed that the shorter segment lacking the *BR-C* binding site was significantly lower than the longer one (Figure 4F). This indicated that *NlFtz-F1* is the target of nlu-miR-173 in response to 20-hydroxyecdysone signaling.

### nlu-miR-173 Is Required for Molting and Chitin Biosynthesis Through *NlFtz-F1*

**Figure 5:**
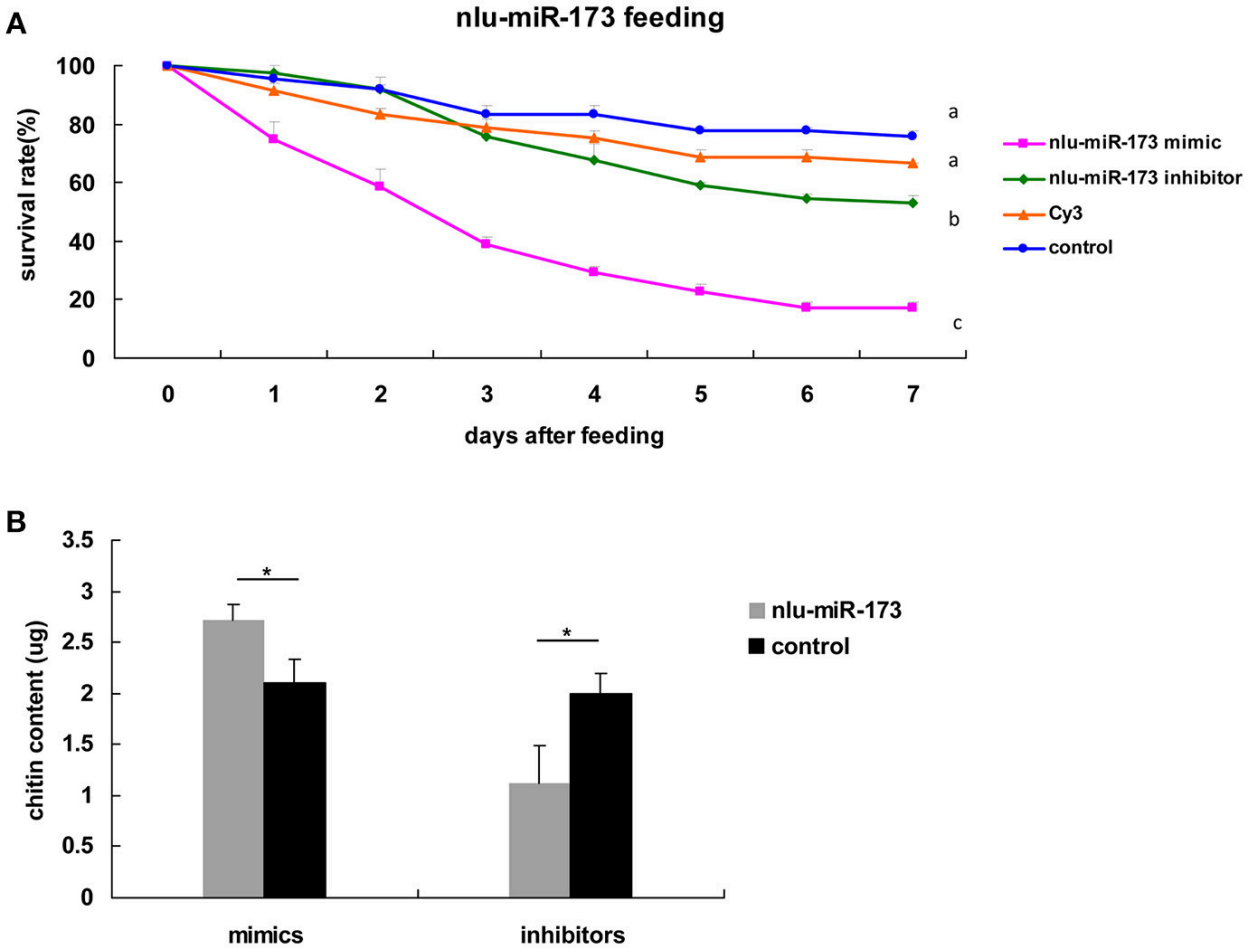
Nlu-miR-173 is required for molting and chitin biosynthesis through *NlFtz-F1*. **(A)** Survival rates with feeding of the miRNA mimics or the inhibitor. Each point represents the mean ± SEM from three independent experiments. Different letters indicate significant differences (*p* < 0.05, LSR, SPSS). **(B)** Mimics or an inhibitor of nlu-miR-173 changed the chitin content of *N. lugens*. A single asterisk indicates a significant difference between the two groups at the *p* < 0.05 level. Each point represents the mean ± SEM (*n* = 3).

**Figure 6:**
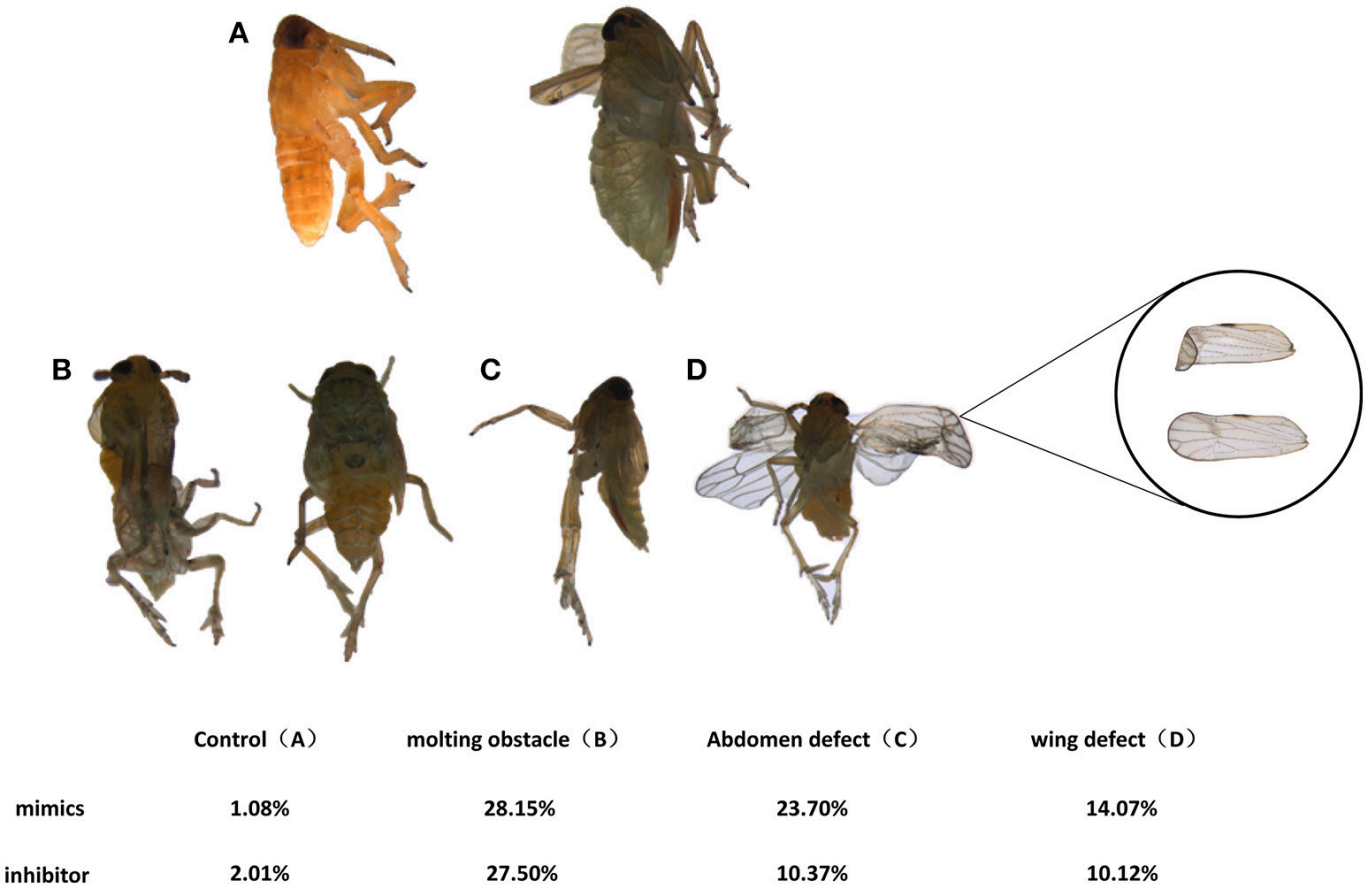
Phenotypes effected by feeding on nymphs. **(A)** Normal adults. Individuals in the mimic-feeding group died with phenotypes of molting failure **(B)**, abdominal defects **(C)** and wing defects **(D)** at rates of 23.70, 28.15, and 14.07%, respectively, whereas individuals in the inhibitor-feeding group died with those phenotypes at rates of 10.37, 27.50, and 10.12%, respectively.

To determine the developmental functions of nlu-miR-173 in *N. lugens, in vivo* nlu-miR-173 mimics or inhibitors were fed on the 3rd nymphs. The death rates of nymphs increased to 84.92 and 47.22% after 7 days of continuous feeding of nlu-miR-173 mimics and inhibitors, respectively, especially a huge mortality of the group of nlu-miR-173 mimics was shown. However, there is no obvious difference between the control and unrelated miRNA (Cy3)-feeding groups (Figure 5A). Individuals fed with nlu-miR-173 mimics or inhibitors exhibited similar phenotypes of molting failure (Figure 6B), abdominal defects (Figure 6C), and wing defects (Figure 6D) at rates of 28.15 and 27.50%, 23.70 and 10.37%, 14.07 and 10.12%, respectively(Figure 6). In addition, the chitin contents changed significantly by nlu-miR-173 mimics or inhibitors injection (Figure 5B). Together, these results indicated that nlu-miR-173 through its target *NlFtz-F1* is required for molting and chitin biosynthesis.

**Figure 7:**
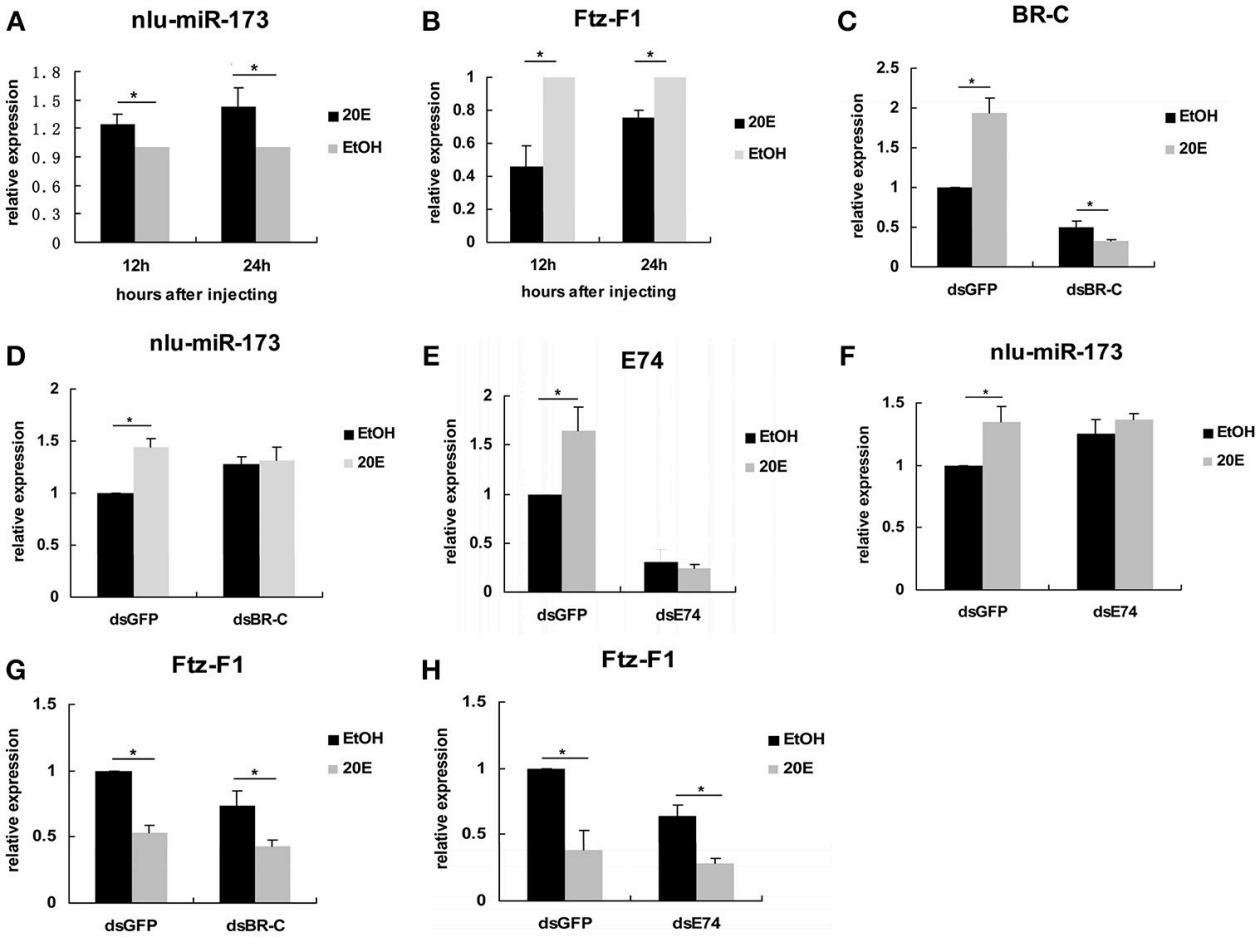
20E signaling regulates nlu-miR-173 through *BR-C* and *E74*. **(A,B)** The expression levels of nlu-miR-173 and Ftz-F1 after 20E injection. **(C–H)** The expression levels of nlu-miR-173, Ftz-F1, BR-C and E74 after 20E and dsRNA injection. All expression levels are shown relative to the levels of *U6* (nlu-miR-173) or β*-actin* (*BR-C, E74*, and *Ftz-F1*), and the expression levels are normalized to those of the ds*GFP*, ds*BR-C*, or ds*E74* group injected with ethanol. A single asterisk indicates a significant difference between the two groups at the *p* < 0.05 level. Each point represents the mean ± SEM (*n* = 3).

To reveal the mechanism that 20E signaling regulated molting and chitin biosynthesis partly through nlu-miR-173 modified by the activity of *BR-C* and *E74* (Ecdysone-induced protein 74EF), we first detected the expression levels of nlu-miR-173 and *NlFtz-F1 in vivo* after 4th-instar nymphs injection by 20E, we found that the expression level of nlu-miR-173 was greatly increased but *NlFtz-F1* was decreased (Figures 7A,B). However, when *BR-C* or *E74* of dsRNAs was injected 24 h before 20E injection, the expression levels of *BR-C, E74*, or nlu-miR-173 was not increased as above (Figures 7C–F), indicating that activity of *BR-C* and *E74* are essential for molting regulated by nlu-miR-173. Further, injection of dsRNAs targeting *BR-C* and *E74* 24 h prior to 20E injection also decreased the expression level of *NlFtz-F1* (Figures 7G,H), showing that *NlFtz-F1* could also be regulated directly by 20E signaling as a transcription factor.

## Discussion

Using the genomic data for *N. lugens* as well as high-throughput sequencing, we have identified candidate miRNAs involved in insect molting. Moreover, we identified the novel miRNA nlu-miR-173 and showed its involvement in the regulation of molting and chitin biosynthesis in *N. lugens* through its target *NlFtz-F1*. This is the first to demonstrate the potential function of miRNAs to act in arthropods at the genome-wide level as regulators of molting and chitin biosynthesis.

### Computational Prediction of miRNAs and their Potential Targets on a Genome-wide Scale

Benefiting from the high-throughput sequencing technology coupled with bioinformatic methods, increasing numbers of miRNAs have been identified many species of insects (Legeai et al., 2010; Liu et al., 2010; Wei et al., 2011; Chilana et al., 2013; Tan et al., 2014). Generally, there are hundreds of known miRNAs in any given insect species, such as *Aedes aegypti, B. mori, D. melanogaster*, and *Tribolium castaneum* with 124, 563, 466, and 430 known mature miRNAs, respectively, according to miRBase 21.0. Recently, a thousand of miRNAs were discovered in *Locusta migratoria* based on both genome searching and small RNA sequencing during different stages and tissues (Wang et al., 2015). In our work, we succeed to found more miRNAs involved in the coordination of molting and chitin biosynthesis by examining stage-specific expression of miRNAs. Based on our previous data and the results in this study, we obtained a total of 326 putative novel miRNAs in *N. lugens*, which significantly extends the list of miRNAs in insect species (Table S6). To predict the putative novel miRNAs, miRNA precursors with hairpin structure and bioinformatic searches based on alignments with the available genome sequences or transcriptomes were performed. Among all previous identified miRNAs in a species, the predicated novel miRNAs usually occupy a small proportion (ranging from 5 to 30%) in most samples (Jagadeeswaran et al., 2010; Wang et al., 2013; Hong et al., 2014; Huang et al., 2014; Zhang et al., 2014b; Hu et al., 2015; Shan et al., 2015; Xu et al., 2015). In previous work, we have acquired 688 conserved and 71 putative novel miRNAs by sequencing from adults and nymphs of *N. lugens* (Chen et al., 2012). However, there was no genome data to obtain the accuracy of miRNA identification in *N. lugens* at that time. Only a little of the 326 putative novel miRNAs identified here overlapped with the previous paper, demonstrating that the quantity of identified miRNAs and the proportion of putative novel miRNAs increased greatly in our work. Moreover, we successfully cloned 36 miRNAs out of 40 selected miRNAs (Figure 2), including 19 conserved and 17 putative novel miRNAs. Considering the limitations of PCR-based methods and the low transcription levels of miRNAs, the predicted rate of false positives would actually be lower than 10%. It showed that our method aboved to predict species-specific miRNAs is referential.

In past few years, it has become routine to predict the targets of all putative miRNAs *in silico* after insect miRNA profiles are reported (Li et al., 2014; Zhang et al., 2014a; Zhou et al., 2014; Jain et al., 2015; Shan et al., 2015; Tariq et al., 2015; Xu et al., 2015; Ling et al., 2017; Wu et al., 2017). However, a large of miRNA targets cannot be verificated by experimental assay. Some studies have reported that only a few miRNAs predicted *in silico* are eventually validated by *in vivo* experiments (Sultan et al., 2014; Suh et al., 2015). False positive and false negative results are unavoidable when conducting genome-wide target prediction due to the precision necessary. We explored the hotmap of miRNAs and their targets in Figure 3, using both miRNA and transcriptome RNA sequencing during molting. It is believed that most miRNAs and their targets appeared a negatively correlation (Lomate et al., 2014). To verify the accuracy of target prediction, a conserved miRNA (miR-34) and a putative novel miRNA (nlu-miR-173) were selected for target validation *in vitro*. We identified *JHBP* and *FTZ-F1* as the respective targets of miR-34 (Figure S2) and nlu-miR-173 (Figure 4) in cells, revealing the high precision of our prediction. Moreover, we also detected a subset of miRNAs that showed both negative and positive correlations with their predicted targets. Recently, some studies has revealed that miRNAs can trigger gene expression via different mechanisms post-transcriptionally, indicating that miRNA and targets also can showed a positive correlation (Vasudevan et al., 2007; Mortensen et al., 2011). This suggests that miRNAs may have different actions toward their targets. However, validation of the relationship between miRNAs and genes showing differential expression between the two molting periods requires more experimental evidence.

### Function of miRNAs in Insect Molting

Recent studies have shown key roles for miRNAs during insect developmental transitions. In *D. melanogaster*, let-7 and miR-34 mutants led to serious metamorphic obstacles (Sempere et al., 2003), and miR-8 may have a key factor in eclosion (Hua et al., 2012). In *B. mori*, let-7 was found its importance in molting and metamorphosis by transgenic miRNA sponge (miR-SP) technology and GAL4/UAS system in silkworm (Lin et al., 2014). Then, CRISPR/Cas9 system mediated miR-14 disruption led to an obvious phenotype in ecdysteriod titers (Liu et al., 2018). In *Bactrocera dorsalis*, ecdysone signaling pathway was triggered by Let-7 through target gene BdE75 (Peng et al., 2017). In *Blattela germanica*, miR-252-3p and miR-2 may play important functions during metamorphosis (Rubio et al., 2012; Jesus et al., 2015). In *N. lugens*, the survival rate and chitin content was affected by miR-8-5p and miR-2a-3p through their targets, membrane-bound trehalase (Tre-2) and phosphoacetylglucosamine mutase (PAGM) (Chen et al., 2013). However, most researchers have focused on this line of research at the level of individual conserved miRNAs. To our knowledge, molting-related miRNAs in insects investigated at the omics level have only been reported in cockroaches, without addressing putative novel miRNAs due to the lack of a complete genome sequence (Rubio et al., 2012). We are the first to detect the molting-related miRNAs on a genome-wide scale, and we identified 19 conserved and 17 putative novel miRNAs with differential expression during molting periods, significantly extending the research on insect molting-related miRNAs.

**Figure 8:**
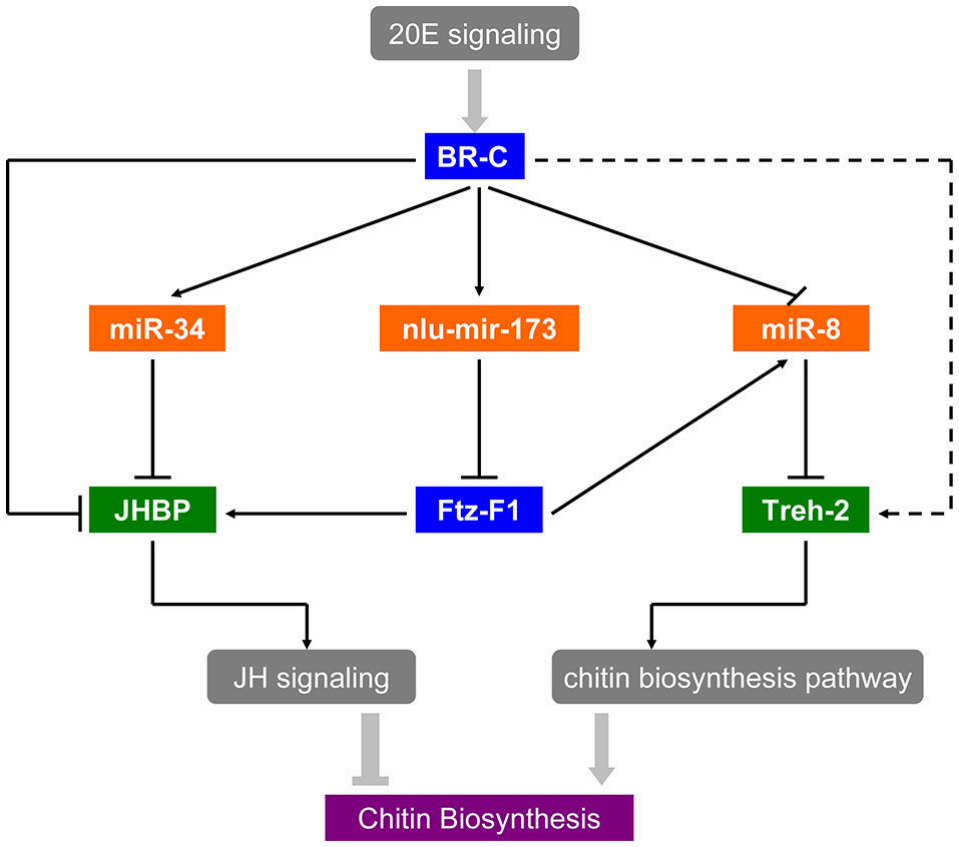
Hormone-miRNA-gene crosstalk in insect chitin biosynthesis. Solid arrows in the picture represent the experimental interactions and broken arrows represent the supposed interactions.

As the interaction between ecdysone and juvenile hormone (JH) is important for molting and metamorphosis (Zhou et al., 1998; Wang et al., 2012), there is accumulating evidence of a link between hormone and miRNAs in insect development and metamorphosis. In *D. melanogaster*, Let-7-Complex can be stimulated by the 20E through *BR-C* whereas miR-34 is repressed by 20E and can be activated by JH (Sempere et al., 2003). Moreover, miR-8-5p and miR-2a-3p are also repressed by *BR-C* and *E74* in *N. lugens* (Chen et al., 2013). Thus, miRNAs can regulate the expression and function of genes in the hormone cascade, for instance, miR-14 via its target *EcR* controls a steroid hormone signaling in *Drosophila* (Varghese and Cohen, 2007), and miR-34 prevents neurodegeneration in the adult brain by repressing *E74A* (Liu et al., 2012). These studies have revealed a regulatory network of miRNAs in the 20E and JH pathways. In *B. mori, BmEcR-B* could be affected by bmo-miR-281 (Jiang et al., 2013). Moreover, *FTZ-F1* and *E74* targeted by Let-7, play crucial factors in the ecdysone pathway (Lin et al., 2014). As shown in our *in vitro* study, miR-34 was activated by BR-C directly because of its bind regions of *BR-C* in the promoter (Figure S2D), and the target gene *JHBP* was down-regulated by miR-34 mimics transfection in S2 cells (Figure S2B), suggested that 20E signaling coordinates with the early response gene *BR-C* to suppress *JHBP* through miR-34. Besides, *JHBP* also could be activated by BR-C directly in Figure S2C. Thus, miR-34 and nlu-miR-173 act as a molecular link that coordinates the two physiological hormone pathways. Further *in vivo* research revealed that the targeting of *NlFTZ-F1* by nlu-miR-173 is a key factor in the action of 20E and acts as a positive regulator of chitin synthesis in *N. lugens*. As a nuclear receptor-type transcription factor, *NlFTZ-F1* could be regulated directly by 20E signaling and suppressed via nlu-miR-173 activated by the 20E. Our present work also reveals that miRNAs and their targets can affect chitin biosynthesis in response to hormone signaling, which reveals hormone-miRNA-gene crosstalk in chitin biosynthesis. In short, there is hormone-miRNA-gene crosstalk that affects chitin biosynthesis and molting (Figure 8).

### Potential Regulatory Mechanism of Ftz-F1 and nlu-miR-173

Mounting evidence suggests that *Ftz-F1* acts importantly responsed by the 20E and JH signaling pathways during molting (Broadus et al., 1999; Bernardo and Dubrovsky, 2012; Cho et al., 2013; Borras-Castells et al., 2017). *Ftz-F1* is induced after the decrease in the 20-hydroxyecdysone level (Woodard et al., 1994; Yamada et al., 2000; Hiruma and Riddiford, 2001), and the function of *Ftz-F1* during metamorphosis in several insects was reported. In *D. melanogaster, Ftz-F1* is required during each stage from embryogenesis, larval ecdysis to pupation (Yamada et al., 2000; Sultan et al., 2014). In several other insect species, silencing *Ftz-F1* caused severe failure of ecdysis or pupation and larval lethality such as *T. castaneum* (Tan and Palli, 2008), *L. Decemlineata* (Liu et al., 2014), and *B. Germanica* (Cruz et al., 2008). Our work is keeping with the finding during the larval period, *Ftz-F1* is induced just before larval ecdysis. The normal expression patterns of nlu-miR-173 and its target *NlFtz-F1* ensure that the molting system operates regularly. Induction of *NlFtz-F1* is prevented by the overexpression of nlu-miR-173, which interrupts the 20E signaling cascade, thereby disrupting the developmental transition, which leads to ecdysis defects and chitin accumulation in *N. lugens*. Our analyses show that nlu-miR-173 is a novel miRNA in *N. lugens*. In *B. mori, Ftz-F1* is a target gene of let-7 (Lin et al., 2014). We need infer that *NlFtz-F1* regulated by nlu-miR-173 may be specific to *N. lugens* or hemimetabolous insects that will be studied in the further. This suggests that let-7 and nlu-miR-173 both target *NlFtz-F1*, indicating that both highly conserved miRNAs and newly evolved miRNAs use this pathway to control development and metamorphosis to achieve common and lineage-specific functions. These results indicate that the comparison of putative novel miRNAs and their targets with conserved miRNAs may help explain the long-term evolution of species, including mechanisms regulating developmental transition in holometabolous and hemimetabolous insects.

## Author Contributions

JC and WZ wrote the paper. JH reviewed the paper. JC and TL did most of the experiments. RP did the bioinformatics works and XY did some of works.

### Conflict of Interest Statement

The authors declare that the research was conducted in the absence of any commercial or financial relationships that could be construed as a potential conflict of interest.

## Supplementary Material

Table S1 All known and novel miRNA sequences identified during molting.

Table S2 All genes differentially expressed in four samples during molting.

Table S3 Targets of all known and putative novel miRNAs in four samples during molting.

Table S4 The expression levels of miRNAs and targets in Figure 3.

Table S5 Primers used in this study.

Table S6 A list of the novel miRNAs in *N. lugens*.

Figure S1 The GO assay of genes differentially expressed during molting. **(A)** cellular component, **(B)** biological process, and **(C)** molecular function. The color scale shows the *p*-value cutoff levels, the more statistically significant, the darker, and redder the color.

Figure S2 Hormone-miRNA-gene crosstalk genes identification. **(A,C,D)** Luciferase assays of miR-8,miR-34, and JHBP. Black and white boxes indicate binding sites for BR-C and Ftz-F1. Double asterisks show a significant difference at the *p* < 0.01 level. The highest luciferase level of a segment is designated as 100%. **(B)** Target identification of miR-34 and JHBP in S2 cells.
