# Supplementary material for: Genome-Wide Screening and Functional Analysis Reveal That the Specific microRNA nlu-miR-173 Regulates Molting by Targeting Ftz-F1 in Nilaparvata lugens
Source: Front Physiol. 2018 Dec 20;9:1854. doi: 10.3389/fphys.2018.01854 (PMC6306441; doi:10.3389/fphys.2018.01854)
Supplement: Table S5 — Primers used in this study. [file Table_5.DOC]

**Table S5 PCR primers used in this study**

| Primers |  | Primer sequence |
| --- | --- | --- |
| For miRNA detection |  |  |
| let-7 |  | 5'-TGAGGTAGTAGGTTGTATAGTA-3' |
| miR-1000 |  | 5'-ATATTGTCCTGTCACAGCAGTA-3' |
| miR-124 |  | 5'-TAAGGCACGCGGTGAATGCCAA-3' |
| miR-133 |  | 5'-TTTGGTCCCCTTCAACCAGCTGT-3' |
| miR-14 |  | 5'-TCAGTCTTTTTCTCTCTCCTATA-3' |
| miR-193 |  | 5'-TACTGGCCTGCTAAGTCCCAAGT-3' |
| miR-1a-5p |  | 5'-CCGTGCTTCCTTACTTCCCATA-3' |
| miR-276-3p |  | 5'-TAGGAACTTCATACCGTGCT-3' |
| miR-2796-5p |  | 5'-AGGGGTTTCTTTCGGCCTCCAG-3' |
| miR-316-5p |  | 5'-TGTCTTTTTCCGCTTTGCTGCCG-3' |
| miR-317 |  | 5'-TGAACACAGCTGGTGGTATCTCAGTT-3' |
| miR-34 |  | 5'-TGGCAGTGTGGTTAGCTGGTTGTG-3' |
| miR-7 |  | 5'-TGGAAGACTAGTGATTTTGTTGTT-3' |
| miR-71-3p |  | 5'-TCTCACTACCTTGTCTTTCATGT-3' |
| miR-750 |  | 5'-CCAGATCTAACTCTTCCAGCTCA-3' |
| miR-87-3p |  | 5'-GTGAGCAAAGTTTCAGGTGTGT-3' |
| miR-8-5p |  | 5'-CATCTTACCGGGCAGCATTAGA-3' |
| miR-92a |  | 5’-TATTGCACTTGTCCCGGCCTA-3’ |
| miR-965 |  | 5’-TAAGCGTATAGCTTTTCCCCTT-3’ |
| miR-993-5p |  | 5’-TACCCTGTAGATCCGGGCTTTT-3’ |
| nlu-miR-3 |  | 5'-TTGGTAACTACTTCACCGATGG-3' |
| nlu-miR-4 |  | 5’-TCGGTAGGCGTGTTACCTCTC-3’ |
| nlu-miR-14 |  | 5’-CTGCTGTATCAGGACATGCCCA-3’ |
| nlu-miR-15 |  | 5’-GGCGAGTGTAAGGCTGGTTCACA-3’ |
| nlu-miR-26 |  | 5’-AGCGAAAGATGAGCTTCAACATTA-3’ |
| nlu-miR-29 |  | 5’-ACGAGTATCATGGGGAAGCTGG-3’ |
| nlu-miR-34 |  | 5’-TGTGATGTTTTTGTGGGTCGTT-3’ |
| nlu-miR-55 |  | 5’-TGTTTGGTCATTGAACGATGCATA-3’ |
| nlu-miR-111 |  | 5’-TAGCACCATAGCATTCAGCTTA-3’ |
| nlu-miR-124 |  | 5’-ACCGGTGGTAGAGGACGGCGGCCG-3’ |
| nlu-miR-135 |  | 5’-CACATAAAGTTGGGTACTGAGGTA-3’ |
| nlu-miR-144 |  | 5’-TATCACAGCCATTTTTGACGTACC-3’ |
| nlu-miR-150 |  | 5’-TAGGAACTTCATACCGTGCTTT-3’ |
| nlu-miR-163 |  | 5’-TGACTAGATCCTTACTCGTCTG-3’ |
| nlu-miR-165 |  | 5’-TAGTACTAGCAGACTGAAGGG-3’ |
| nlu-miR-173 |  | 5’-TGCTTGGTAGATGCTTGTAGG-3’ |
| nlu-miR-186 |  | 5’-TTAATGTCACTCCATGTAGCGGT-3’ |
| nlu-miR-213 |  | 5’-TATTGATTTGTCCACTCTGATTG-3’ |
| nlu-miR-244 |  | 5’-GCTAAGGAGATTCCACGACATTT-3’ |
| nlu-miR-282 |  | 5’-GAATTTATTCAGTCTGGTACATTT-3’ |
| mRQ3' |  | 5’-AAGCAGTGGTATCAACGCAGAGTAC-3’ |
|  |  |  |
| For vector constructs |  |  |
| 3’-FTZ-F |  | 5’-gctctagaCATCTTGGACCACGCAATCA-3’ |
| 3’-FTZ-R |  | 5’-gctctagaGTTTGCTTGCAACTACTGGTAGG-3’ |
|  |  |  |
| For dsRNA synthesis |  |  |
| dsBR-C-F |  | 5'-ATGGGGACTACTCAGCAGTTT-3' |
| dsBR-C-R |  | 5'-TTCAGGTTGTTCAGGTGTTGAC-3' |
| dsE74-F |  | 5’-CAGTTGTCGTCGGAGGGATGGA-3’ |
| dsE74-R |  | 5’-TGCTCGTGACCTTGGCTGTGAT-3’ |
| dsGFP-F |  | 5’-AAGGGCGAGGAGCTGTTCACCG-3’ |
| dsGFP-R |  | 5’-CAGCAGGACCATGTGATCGCGC-3’ |
|  |  |  |
| For real-time PCR |  |  |
| QBR-C-F |  | 5'-GATCATCGTGGTCTCAATTCCT-3' |
| QBR-C-R |  | 5'-CGCCGTTGTGCCTATTCTT-3' |
| QFtz-F |  | 5'-CAGTTGTCGTCGGAGGGATGGA-3' |
| QFtz-R |  | 5'-TGCTCGTGACCTTGGCTGTGAT-3' |
| QE74-F |  | 5'-ACGACATATCTGTGGGAATTTCTG-3' |
| QE74-R |  | 5'-GGTCTCGTAGTTCATGTCAGGCT-3' |
| QACTIN-F |  | 5'-TGCGTGACATCAAGGAGAAGC-3' |
| QACTIN-R |  | 5'-CCATACCCAAGAAGGAAGGCT-3' |

F: Forward; R: Revers
