# Supplementary figures and images for: Genome-Wide Screening and Functional Analysis Reveal That the Specific microRNA nlu-miR-173 Regulates Molting by Targeting Ftz-F1 in Nilaparvata lugens
Source: Front Physiol. 2018 Dec 20;9:1854. doi: 10.3389/fphys.2018.01854 (PMC6306441; doi:10.3389/fphys.2018.01854)

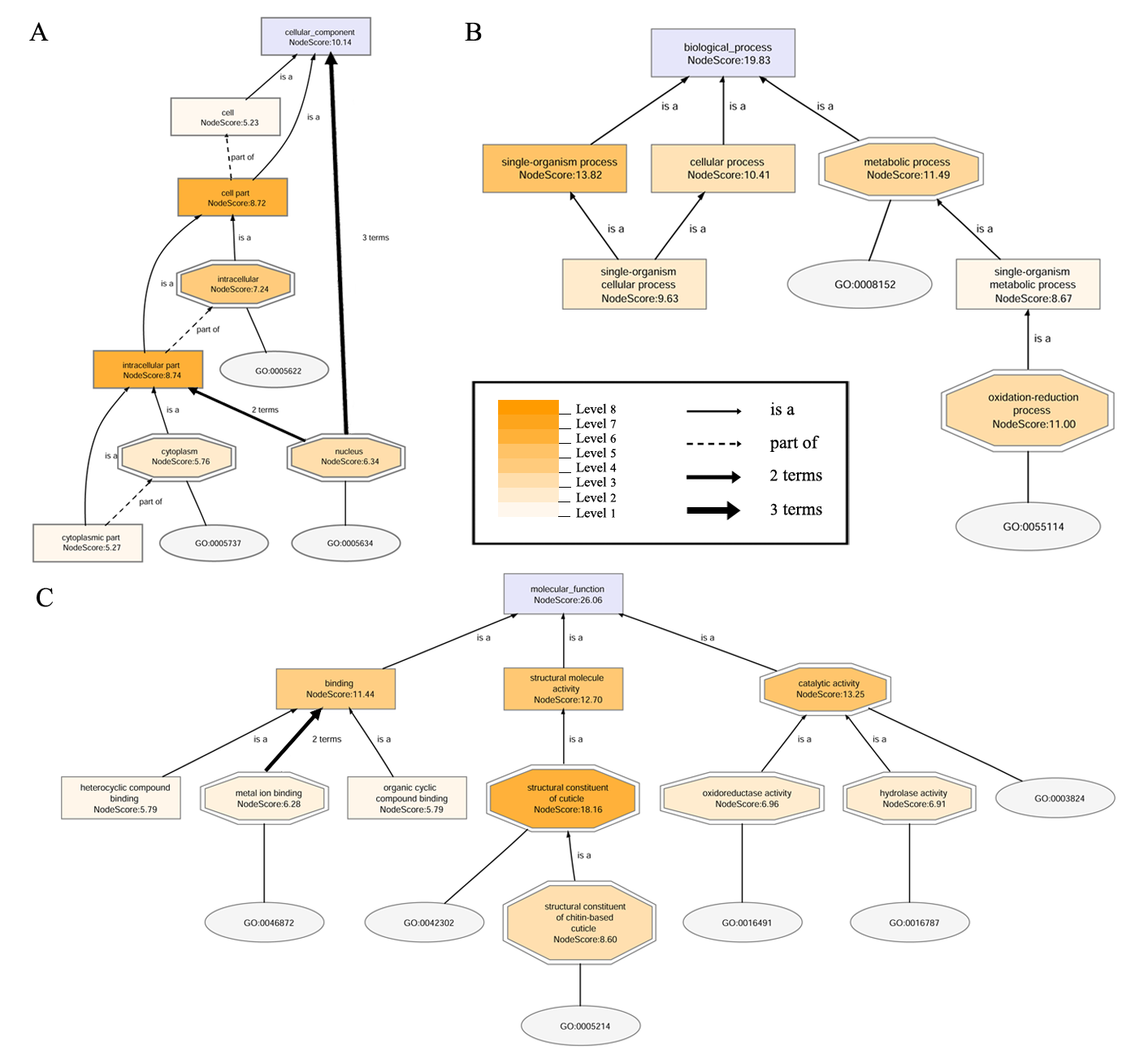

Supplement: Figure S1 — The GO assay of genes differentially expressed during molting. (A) cellular component, (B) biological process, and (C) molecular function. The color scale shows the p-value cutoff levels, the more statistically significant, the darker, and redder the color. [file Image_1.TIF]

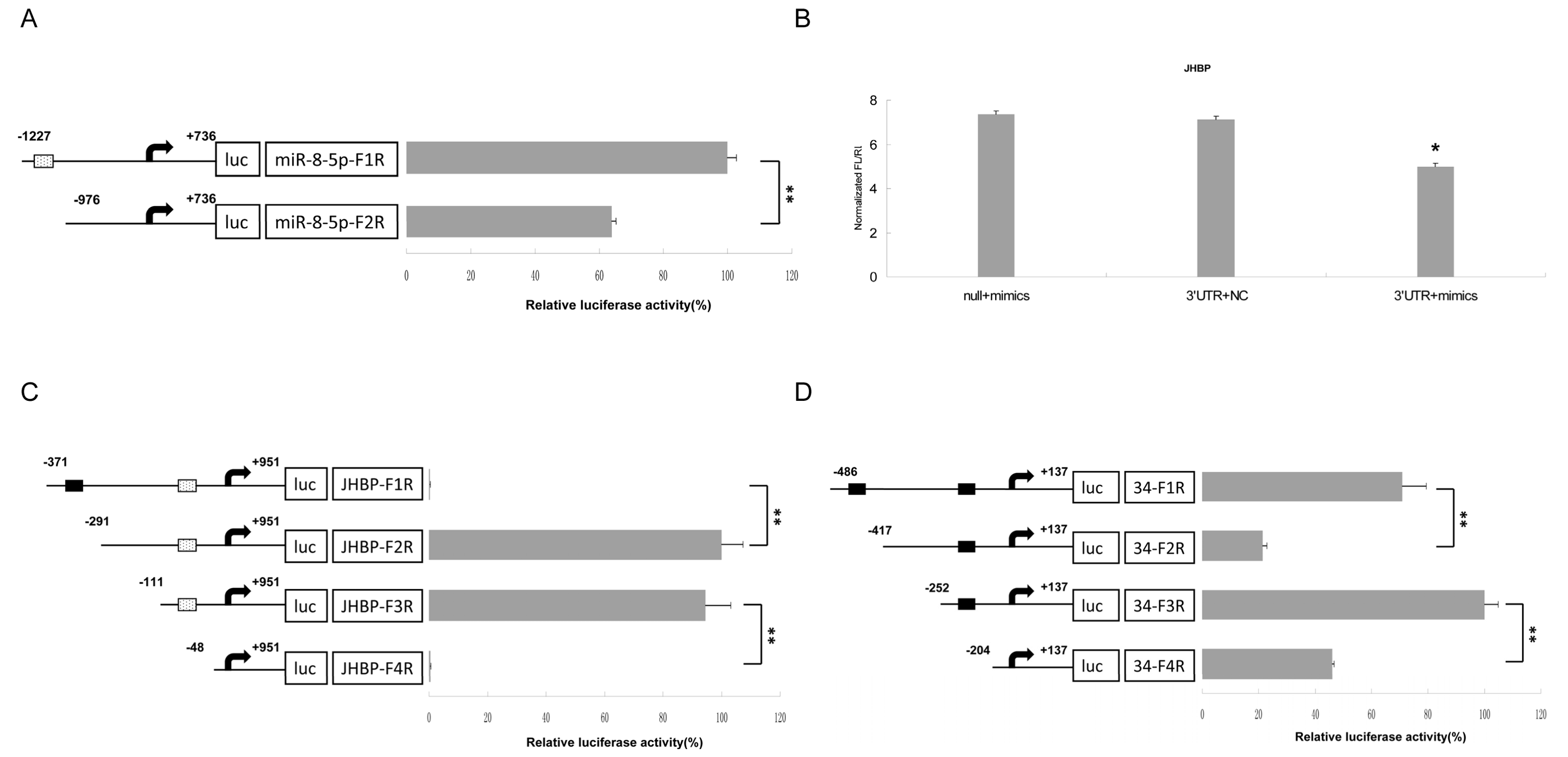

Supplement: Figure S2 — Hormone-miRNA-gene crosstalk genes identification. (A,C,D) Luciferase assays of miR-8,miR-34, and JHBP. Black and white boxes indicate binding sites for BR-C and Ftz-F1. Double asterisks show a significant difference at the p < 0.01 level. The highest luciferase level of a segment is designated as 100%. (B) Target identification of miR-34 and JHBP in S2 cells. [file Image_2.TIF]
